# *β*-Naphthoflavone-Induced Mitochondrial Respiratory Damage in Cyp1 Knockout Mouse and in Cell Culture Systems: Attenuation by Resveratrol Treatment

**DOI:** 10.1155/2017/5213186

**Published:** 2017-09-14

**Authors:** Suresh Kumar Anandasadagopan, Naveen M. Singh, Haider Raza, Seema Bansal, Venkatesh Selvaraj, Shilpee Singh, Anindya Roy Chowdhury, Nicolae Adrian Leu, Narayan G. Avadhani

**Affiliations:** ^1^Department of Biomedical Sciences, School of Veterinary Medicine, University of Pennsylvania, Philadelphia, PA 19104-6009, USA; ^2^Department of Biochemistry, College of Medicine and Health Sciences, United Arab Emirates University, Al-Ain, UAE

## Abstract

A number of xenobiotic-inducible cytochrome P450s (CYPs) are now known to be localized in the mitochondrial compartment, though their pharmacological or toxicological roles remain unclear. Here, we show that BNF treatment markedly inhibits liver mitochondrial O_2_ consumption rate (OCR), ADP-dependent OCR, and also reserve OCR, in wild-type mice but not in *Cyp1a1/1a2(−/−)* double knockout mice. BNF treatment markedly affected mitochondrial complex I and complex IV activities and also attenuated mitochondrial gene expression. Furthermore, under in vitro conditions, BNF treatment induced cellular ROS production, which was inhibited by mitochondria-targeted antioxidant Mito-CP and CYP inhibitor proadefin, suggesting that most of the ROS production was intramitochondrial and probably involved the catalytic activity of mitochondrial CYP1 enzymes. Interestingly, our results also show that the AHR antagonist resveratrol, markedly attenuated BNF-induced liver mitochondrial defects in wild-type mice, confirming the role of AHR and AHR-regulated CYP1 genes in eliciting mitochondrial dysfunction. These results are consistent with reduced BNF-induced mitochondrial toxicity in *Cyp1a1/1a2(−/−)* mice and elevated ROS production in COS cells stably expressing CYP1A1. We propose that increased mitochondrial ROS production and respiratory dysfunction are part of xenobiotic toxicity. Resveratrol, a chemopreventive agent, renders protection against BNF-induced toxicity.

## 1. Introduction

Studies from our laboratory as well as from others have shown that xenobiotic-inducible CYPs (CYP1A1, 1A2, 1B1, 2E1, 2B1, 2C8, and 3A4/5), as well as the constitutively expressed CYP2D6, are also targeted to mitochondria where they actively catalyze substrate oxidation in association with adrenodoxin (ADX) and adrenodoxin reductase (ADR). In several cases, these mitochondria-localized CYPs exhibit altered substrate specificity [1–6]. Recent studies also suggest that aryl hydrocarbon receptor (AHR), a ligand-activated basic helix-loop-helix (bHLH) transcription factor [7] is also localized in the mitochondrial intermembrane space [8], although its role in mitochondrial gene expression or biogenesis remains unclear. AHR ligands in the cytoplasmic compartment include polycyclic aromatic hydrocarbons (PAHs) and halogenated aromatic hydrocarbons (including 2,3,7,8-tetrachloro-dibenzo-p-dioxin,TCDD). In addition, a large number of as-yet-unidentified endogenous compounds also activate AHR [7, 9, 10].

PAHs induce transcriptional activation of the *Cyp1* (*CYP1a1, 1a2*, and *1b1*) gene family in addition to induction of an array of phase II drug-detoxifying enzymes such as glutathione transferases and glucuronyl transferases [11–14]. CYP1A1, 1A2, and 1B1 are also major enzymes which metabolize and eliminate PAHs [15–17] that are components of cigarette smoke, cooked food, and industrial pollution. All three CYP1 family members are induced abundantly in the lung, skin, brain, intestine, and bone marrow cells in response to exposure to AHR agonists, whereas in the mammalian liver only 1A1 and 1A2 are induced [9, 16, 18, 19]. In a recent study, we showed that BaP treatment induced lung mitochondrial dysfunction including reduced respiratory capacity, altered cytochrome c oxidase (CcO) activity, and decreased mtDNA levels. Transgenic *Cyp1a1/1a2* (−/−) double knockout and *Cyp1b1−/−* mice were relatively resistant to BaP-induced mitochondrial toxicity [20]. Furthermore, shRNA-mediated knockdown of NADPH-cytochrome P450 oxidoreductase (NPOR) and ADX mRNA suggested that mitochondrial CYP1A1 and 1B1-dependent metabolism play a role in BaP-induced lung mitochondrial dysfunction [20].

Previous studies by Senft et al. [21, 22] showed that TCDD induces ROS production and mitochondrial respiratory defects possibly through AHR activation mechanism. A recent study showed that TCDD induces degradation of mitochondrial AHR in a manner similar to the nuclear/cytoplasmic AHR [8]. Studies in our laboratory showed that TCDD imparts both AHR-dependent and independent effects on mitochondrial function and nuclear gene expression [23]. Specifically, we showed that TCDD induces mitochondrial dysfunction and retrograde signaling which is likely due to the direct action of xenobiotic on mitochondrial inner membrane rather than through AHR activation [23].

We therefore hypothesized that BaP-mediated mitochondrial dysfunction could be attributed to two possible mechanisms: (a) CYP1 monooxygenase activity might directly or indirectly contribute to oxidative stress affecting mitochondrial function, or (b) resulting reactive oxygen species (ROS) and metabolites could damage mtDNA or mitochondrial integrity leading to mitochondrial dysfunction. In this study, therefore, using transgenic *Cyp* double (Cyp1a1/1a2) or triple (Cyp1a1/1a2/1b1) KO mice, in vivo and C6 glioma cell culture in vitro, we investigated the effects of *β*-naphthoflavone (BNF), a known inducer of CYP1A1/1A2 and an AHR agonist, whose metabolic products, unlike other PAHs like BaP, are neither reactive nor toxic. Our results show that BNF induces oxidative stress and mitochondrial dysfunction similar to that observed in BaP-treated lung mitochondria [3, 24]. Furthermore, the BNF-induced toxicity is probably due to CYP1 enzyme-mediated ROS production which is attenuated by resveratrol, a naturally occurring polyphenolic compound, using both in vivo and in vitro model systems. In addition, we have also demonstrated that mitochondria-targeted antioxidant, Mito-CP, and CYP inhibitor proadefin also inhibit BNF-induced mitochondrial dysfunction suggesting the role of oxidative stress and CYP catalytic activity in BNF-induced toxicity.

## 2. Materials and Methods

### 2.1. Experimental Models

We used Wt and Cyp1a1/1a2(−/−) and Cyp1a1/1a2/1b1(−/−) mice to evaluate the roles of these CYPs in BNF-induced effects on liver mitochondrial respiration and electron transport chain complex activities. To evaluate the generality of BNF-mediated effects on mitochondrial function and ROS production, we used C6 glioma, a neuroglial cell line. COS-7 cell lines stably expressing full-length CYP1A1 and N-terminal truncated +331A1, which is preferentially targeted to the mitochondria ([1]; Dasari et al. 2006), were used to evaluate the role of CYP1A1 catalytic activity in ROS production. Also, use of these cell lines allowed the facile evaluation of different pharmacological agents on BNF-mediated effects.

### 2.2. Reagents and Cell Cultures

*β*-Naphthoflavone (BNF), DMSO, catalase, ubiquinol, ADP, sodium succinate, NADH, cytochrome C, lauryl maltoside, oligomycin, 2,4-dinitrophenol (DNP), rotenone, antimycin, CH223191, proadifen, and resveratrol were obtained from Sigma Chemical Co. (St Louis, MO). ROS probes 2′,7′–dichlorofluorescin diacetate (DCFDA) and Amplex Red reagents were purchased from Abcam (Cambridge, MA) and Invitrogen, (Carlsbad, CA), respectively. Rat C6 glioma and COS cells were purchased from American Type Culture Collection (ATCC) (Manassas, VA) and grown in DMEM/F12 or MDM2 media obtained from Invitrogen, (Carlsbad, CA). In all cases, cells were grown in culture medium supplemented with 10% fetal bovine serum, 1% penicillin-streptomycin at 5% CO_2_, and 95% air (v/v), at 37°C in the incubator. In some cases, cells were also treated for 24–48 hrs with BNF dissolved in dimethylsulfoxide (DMSO; 25–50 *μ*M) in the presence or absence of resveratrol (10 *μ*M), Mito-CP (2 *μ*M), AHR inhibitor CH223191 (25 *μ*M), and CYP inhibitor proadifen (5 *μ*M), whereas the control cells were treated with vehicle alone.

### 2.3. Animal Studies

Wild type (Wt), *Cyp1a1/1a2(−/−)* double KO, and *Cyp1a1/1a2/1b1(−/−)* triple KO mice were obtained from the Daniel Nebert's mouse colony (University of Cincinnati Medical Center). Male mice aged (6–8 weeks) were divided into three different groups (*n* = 4 − 5 each). Group I (controls) received intraperitoneal (i.p.) corn oil as vehicle control. Group II animals were treated intraperitoneal (IP) with BNF alone (50 mg/kg body weight) in corn oil for 7 consecutive days. Group III mice were treated IP with BNF (50 mg/kg body weight) plus resveratrol (20 mg/kg body weight). The dosage and time points for BNF treatment *in vivo* were based on previous literature and our own published studies [25, 26], and the resveratrol dose was based on [27, 28]. Mice were euthanized by CO_2_ asphyxiation protocol using a Crainey Tech asphyxiation chamber in accordance with the American Veterinary Medical Association (AVMA) and National Institutes of Health (NIH) approved guidelines. The livers from control and treated mice were collected and used for preparing subcellular fractions for further studies and extraction of total RNA or total DNA.

### 2.4. Preparation of Mitochondrial Extracts

The livers were perfused and rinsed with phosphate-buffered saline and homogenized in a motor-driven glass-Teflon homogenizer in H-medium (70 mM sucrose, 220 mM mannitol, 2.5 mM Hepes, pH 7.4, 2 mM EDTA, and complete protease inhibitor mixture). Mitochondria and microsomes from freshly extracted mouse liver were prepared by differential centrifugation and suspended in 20 mM K_2_HPO_4_ buffer containing 20% glycerol with added leupeptin, pepstatin, antipain, and PMSF as described previously [29, 30]. Treatment with protease inhibitor was excluded when mitochondria or microsomes were used for enzyme assays or respiratory measurements. Protein concentration of cell fractions was determined by the method of Lowry et al. [31].

### 2.5. Measurement of Mitochondrial Electron Transport Enzyme Activity

Complex I (NADH: ubiquinone oxidoreductase) and complex IV (cytochrome c oxidase (CcO)) activities were measured according to the method of Birch-Machin and Turnbull [32]. Briefly, complex I assay was carried out, by incubating 15 *μ*g of freeze-thawed mitochondrial extract in 1 mL of assay medium (25 mM potassium phosphate, pH 7.4, 5 mM MgCl_2_, 2 mM NaCN, 2.5 mg/ml bovine serum albumin, 13 mM NADH, 65 *μ*M ubiquinone, and 2 *μ*g/ml antimycin A), and then measuring the decrease in absorbance at 340 nm due to NADH oxidation using a Cary 1E UV-visible spectrophotometer. Rotenone-sensitive complex I activity was measured by addition of 40 *μ*M rotenone. Complex IV (CcO) activity was measured by incubating 2–10 *μ*g of freeze-thawed mitochondrial extract in 1 mL of assay medium (25 mM potassium phosphate, pH 7.4, and 0.45 mM dodecylmaltoside). Ferrocytochrome c (15 *μ*M) was added, and reaction rates were measured using a Cary 1E spectrophotometer. First-order rate constants were calculated based on regression analysis, using the Cary-Win kinetics software. The molar extinction coefficient (*€*) of 21.1 was used for the conversion of OD units to molar amounts of reduced cytochrome c oxidized as described before [20, 33].

### 2.6. Measurement of Mitochondrial Respiration

Seahorse XF24 Extracellular Flux Analyzer (Seahorse Biosciences, North Billerica, MA, USA) was used to assess mitochondrial respiratory function. Freshly isolated mitochondria (10 *μ*g/well) from control and treated mouse livers were plated onto Seahorse poly-L-lysine plates. Mitochondria were energized by adding 8 mM succinate. Respiration was sequentially measured in energized mitochondria (basal respiration), followed by state 3 (phosphorylating respiration, in the presence of 5 mM ADP), and state 4o (resting respiration) with the addition of 3 *μ*g/mL oligomycin, an inhibitor of mitochondrial ATPase. Uncoupled maximal respiration (state 3u) was determined by the administration of 150 *μ*M DNP. Finally, for complete inhibition of mitochondrial respiration, complex III inhibitor antimycin A (4 *μ*g/ml) was added. Results were plotted as percentage value of state 3 respiration of respective control, set as 100%.

### 2.7. Measurement of ROS Formation

ROS formation was measured, using DCFDA or Amplex Red as probes, according to manufacturer suggested protocols described before [20, 34]. Briefly, 10–20 *μ*g of mouse liver mitochondria from control and BNF-treated mice were incubated in mitochondrial buffer (H-medium) pH 7.4 in the presence of 1 *μ*g of sodium dodecylmaltoside and 20 *μ*M NADH with or without 2 *μ*g/mL catalase followed by 10 min incubation at 37°C. For measuring cellular ROS in untreated and treated MCF-7 and A549 cells, the DCFDA method was used as described above. ROS formation was measured using the LPS-220B Photon Technology International Fluorescence Instrument (Birmingham, NJ). For DCFDA assay, we measured ROS formation at excitation wavelength 485 nm and emission wavelength 535 nm or for the Amplex Red assay at excitation wavelength 530 nm and emission 590 nm, and probes using MicroWin chameleon multilabel detection platform were used. Membrane permeable SOD was used as a control to confirm H_2_O_2_ production.

### 2.8. Quantification of mRNA

Total RNA was isolated from fresh liver slices or cell pellets using TRIzol reagent (Invitrogen) using the manufacturer's recommended protocol. For real-time PCR analysis, RNA was digested with turbo DNase I (Ambion, Inc.), and cDNA was synthesized using 1 *μ*g total RNA as template by using the High-Capacity cDNA Archive kit (Applied Biosystems, Inc.). Relative mRNA levels of CYP1A1, CYP1A2, and CYP1B1 were measured by standard SYBR Green real-time PCRs on an ABI 7300 real-time PCR machine as described before [29]. Transcript levels were normalized to the housekeeping gene *β*-actin (ACTB) as an internal control and expressed as % change relative to ACTB mRNA.

### 2.9. Preparation of COS Cells Stably Expressing CYP1A1 Protein

Full-length and N-terminal truncated +331A1 cDNAs were cloned in BamH1 and XBA-1 sites of pGP Lenti (Addgene, Cambridge, MA) vector, and fully assembled viral particles were generated by transfection in 293T cells. To generate stable lines, COS-7 cells were transduced with Lenti viral constructs as per the vendor's protocol. Transduced cells were selected based on resistance to 2 *μ*g/mL puromycin (Invitrogen, Carlsbad, CA). Fresh puromycin-containing media was exchanged every other day for ~2 weeks. After 8–10 days, individual colonies were selected and trypsinized using 0.05% Trypsin EDTA (Thermo Fisher, Waltham, MA) and transferred to a new six-well plate for another 2–4 days or until the cell density reached 50–70% confluence. Monoclonal selection was achieved by means of serial dilution to 2 cells/mL and plated at 100 *μ*L DMEM F12 puromycin-containing media per well in a 96-well plate. Wells contained colonies derived from a single cell were selected and expanded into a 24-well plate. Protein levels were measured by immunoblotting with an anti–CYP1A1 antibody (in house generated [2]).

### 2.10. SDS-PAGE and Immunoblot Analysis

For SDS-PAGE analysis, mitochondrial protein (50 *μ*g) was separated on 12% SDS-polyacrylamide gels. Proteins were then transferred to nitrocellulose membranes (Bio-Rad) and probed with the appropriate antibodies. Antibodies specific to CcO I (cat. number ab14705), CcO IVi1 (cat. number ab110272), CcO Vb (cat. number ab110263), succinate dehydrogenase complex flavoprotein subunit A (SDHA) (cat. number ab14715), and voltage-dependent anion channel (VDAC) (cat. number ab61273) were obtained from Abcam (Cambridge, MA). Antibodies for CYP1A2 (cat. number sc-30,085), NADPH-P450 oxidoreductase (NPOR) (cat. number sc-25,270), and *β*-actin (cat. number sc-1616) were purchased from Santa Cruz Biotechnology, Inc. (Dallas, TX). Blots were probed with appropriate dilution of primary (concentration~ 1 *μ*g/ml) followed by corresponding secondary antibodies (manufacturer recommended concentration). The blots were developed using the SuperSignal West Femto System (Pierce) and imaged on a Bio-Rad VersaDoc Imaging System or an Odyssey Licor (Licor Biotechnology, Lincoln, NE). Digital image analysis was performed using Quantity One Version 4.5 software from Bio-Rad.

The blue native gel electrophoresis protocol (BNGE) for the separation respiratory complexes was as described before [35]. Mitochondrial membrane complexes solubilized in 1% laurylmaltoside were clarified at 100,000 ×g for 30 min and mixed with 5% Serva blue dye. BNGE was performed on a native 6–13% polyacrylamide gradient gel starting with a current of 100 V and increasing to a constant current of 250 V. Individual complexes were identified based on the size and reactivity to antibodies [35].

### 2.11. Statistical Analysis

The means ± SEM were calculated from three to five independent experimental values. Statistical significance (*p* values) between control and experimental or paired experiments were calculated using Student's *t*-test. A *p* value of ≤0.05 was considered significant.

## 3. Results

### 3.1. BNF-Induced Mitochondrial Respiratory Defects and the Effects of Resveratrol Treatment

**Figure 1:**
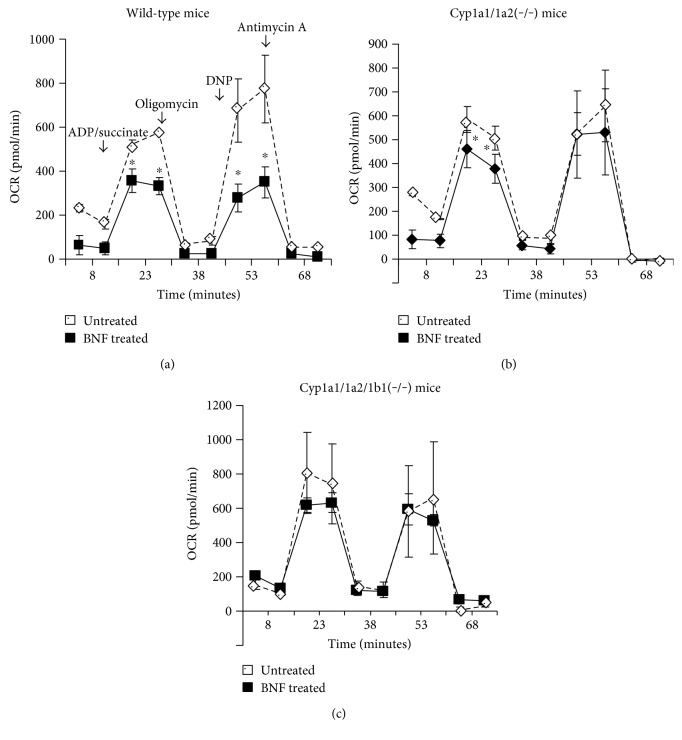
Effect of BNF and resveratrol treatment on mitochondrial respiration in wild-type, double *Cyp1a1/1a2(−/−)*, and triple *Cyp1a1/1a2/1b1(−/−)* knockout mice. Oxygen consumption rate (OCR) in mouse (*n* = 4) liver mitochondria isolated from wild-type (a), double knockout *Cyp1a1/1a2(−/−)* (b), and triple knockout *Cyp1a1/1a2/1b1(−/−)* mice (c) treated with BNF was monitored through Seahorse XF-24 Extracellular Flux Analyzer as described in Materials and Methods. Baseline, ATP synthesis, proton leak, and spare respiratory capacity are presented as means ± SEM for at least 4 independent experiments. ^∗^*p* < 0.05 versus respective untreated and BNF-treated groups of mice.

We compared the effects of BNF and resveratrol treatment on respiration profiles of wild-type mice as well as the double (*Cyp1a1/1a2^−/−^)* and triple knockout (*Cyp1a1/1a2/1b1^−/−^*) groups of mice. The rationale was to see if BNF, a relatively nontoxic AHR agonist, induced respiratory dysfunction in the liver and if the toxicity was associated with the expression of CYP1A1/1A2/1B1 proteins. We measured the respiratory parameters including the baseline OCR, OCR linked to ATP synthesis, maximal respiration, and the state 3 respiration. Results show that BNF treatment markedly reduced the maximal respiration (after addition of DNP) as well as the reserve respiration in Wt mice but had no significant effect in *Cyp1a1/1a2^−/−^* and *Cyp1a1/1a2/1b1^−/−^* triple knockout mice (Figures 1(a), 1(b), and 1(c)). These results suggest that BNF induced respiratory impairment in Wt mice that was likely associated with the expression of CYP1 and/or CYP1A2 proteins.

As shown in Figure 1, the average baseline OCR for wild-type liver mitochondria seeded at 10 *μ*g/well was 230 pmol O_2_/min, including nonmitochondrial oxygen consumption, which was determined after the addition of antimycin, a potent inhibitor of complex III which channels electrons from both NADH (complex I) and FADH2 (succinate) (complex II). The portion of mitochondrial oxygen consumption devoted to ATP synthesis (which is blocked by oligomycin) was ~70%, while the remaining ~30% fed into proton leak. Maximal respiratory rate was determined after addition of DNP, an ionophore which transfers protons across the membrane independently of ATP synthesis and reveals the full potential for O_2_ consumption. The difference between maximal and baseline OCR is defined as reserve respiratory capacity. Our results show that the hepatic mitochondrial oxygen consumption is markedly inhibited by BNF in wild-type mice (Figure 1(a)); however, no significant inhibition was observed in double (*Cyp1a1/1a2^−/−^)* (Figure 1(a)) and triple knockout (*Cyp1a1/1a2/1b1^−/−^*) (Figure 1(c)) groups of mice.

**Figure 2:**
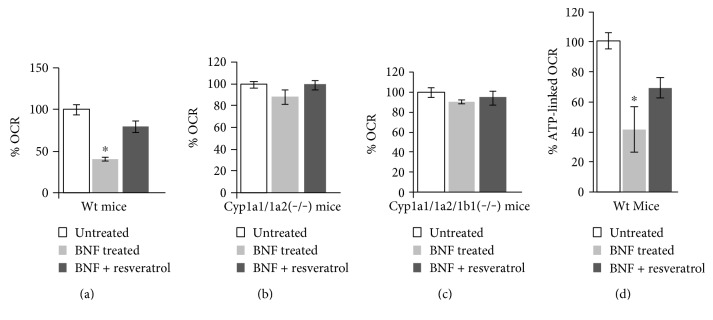
Quantitation of state 3 mitochondrial respiration in BNF-treated wild-type and knockout mice. State 3 respiration capacity was measured in mouse liver mitochondria isolated from BNF- and resveratrol-treated wild-type (a), *Cyp1a1/1a2(−/−)* (b), and triple *Cyp1a1/1a2/1b1(−/−)* (c) knockout mice (*n* = 4 each) along with nontreated control mice as in Figure 1. Changes in % ATP-linked OCR (d) were measured using XF24 metabolic analyzer. Values are presented as means ± SEM of at least 4 independent measurements for each mouse. ^∗^*p* < 0.05 versus respective wild-type control, BNF, and resveratrol treatment.

Mitochondria from Wt mice treated with BNF showed 45% reduction in state 3 respiration as compared with 100% for mitochondria from untreated mice. As seen from Figure 2(a), administration of resveratrol alleviated BNF-induced reduction in state 3 OCR. The state 3 respiration was minimally affected in the double (Figure 2(b)) and triple knockout mice (Figure 2(c)) by BNF treatment, and as expected, resveratrol had no significant effect on the respiration. Consistent with this, BNF treatment in wild-type mice resulted in ~50% decrease in ATP-linked OCR which was restored to 70% after resveratrol treatment (Figure 2(d)). These results show that BNF treatment affected mitochondrial respiratory controls in a *CYP1* gene dependent manner, and the AHR antagonist resveratrol had a protective effect on BNF-induced respiratory defects.

### 3.2. Effects of BNF and Resveratrol on Liver Mitochondrial Electron Transport Complex Activities

**Figure 3:**
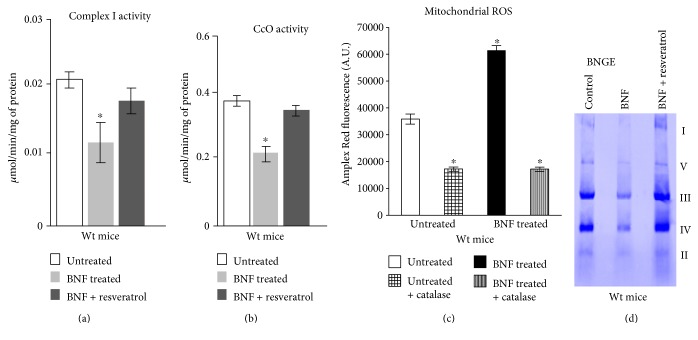
Effects of BNF and resveratrol on mitochondrial function and ROS production in wild-type mouse liver. Complex I activity (a) and CCo activity (b) were measured in mitochondria isolated from control, BNF-, and resveratrol-treated groups (*n* = 4 each). ROS formation was measured in control and BNF-treated mouse liver mitochondria by Amplex Red assay system (c) using MicroWin chameleon multilabel detection platform at excitation wavelength 530 nm and emission 590 nm as described in materials and methods. (d) Blue Native Gel resolution of mitochondrial complexes from livers of control (untreated), BNF-treated and BNF plus resveratrol-treated mice. The pattern was repeated three times using extracts (80 *μ*g each) from three separate mice for each group. Details were as described in Materials and Methods. Results represent mean ± SEM from 4 independent assays. ∗ denotes *p* < 0.05.

We investigated the effects of BNF on complex I activity, which is a major contributor of ROS production in the mitochondrial compartment, and complex IV activity, which is the terminal oxidase of the mitochondrial electron transport chain. The results show that complex I activity was significantly inhibited (~45%) in the BNF-treated liver mitochondria as compared with control livers and resveratrol treatment restored the activity close to the untreated control level (Figure 3(a)). We also observed steady decrease (~40%) in CcO activity in the livers of mice treated with BNF as compared with control (Figure 3(b)). Resveratrol treatment brought both complex I and IV activities close to controls (Figures 3(a) and 3(b)).  Figure 3(c) shows that mitochondrial ROS production in mouse liver mitochondria, as measured by Amplex Red increased (~1.7 fold of control) after BNF treatment. Catalase treatment in both BNF-treated and untreated liver mitochondria attenuated the ROS production suggesting that the increased fluorescence signal observed with BNF treatment was indeed due to increased H_2_O_2_ production. The BNGE pattern of mitochondrial complexes in Figure 3(d) shows that the relative band intensities for complex I, III, and IV were significantly lower in mitochondria from BNF-treated mice (48 h) that was attenuated by coadministration of resveratrol. These results are consistent with the enzyme activity data presented in Figures 3(a) and 3(b).

### 3.3. Effects of BNF and Resveratrol on the Steady State Levels of CcO Subunits

**Figure 4:**
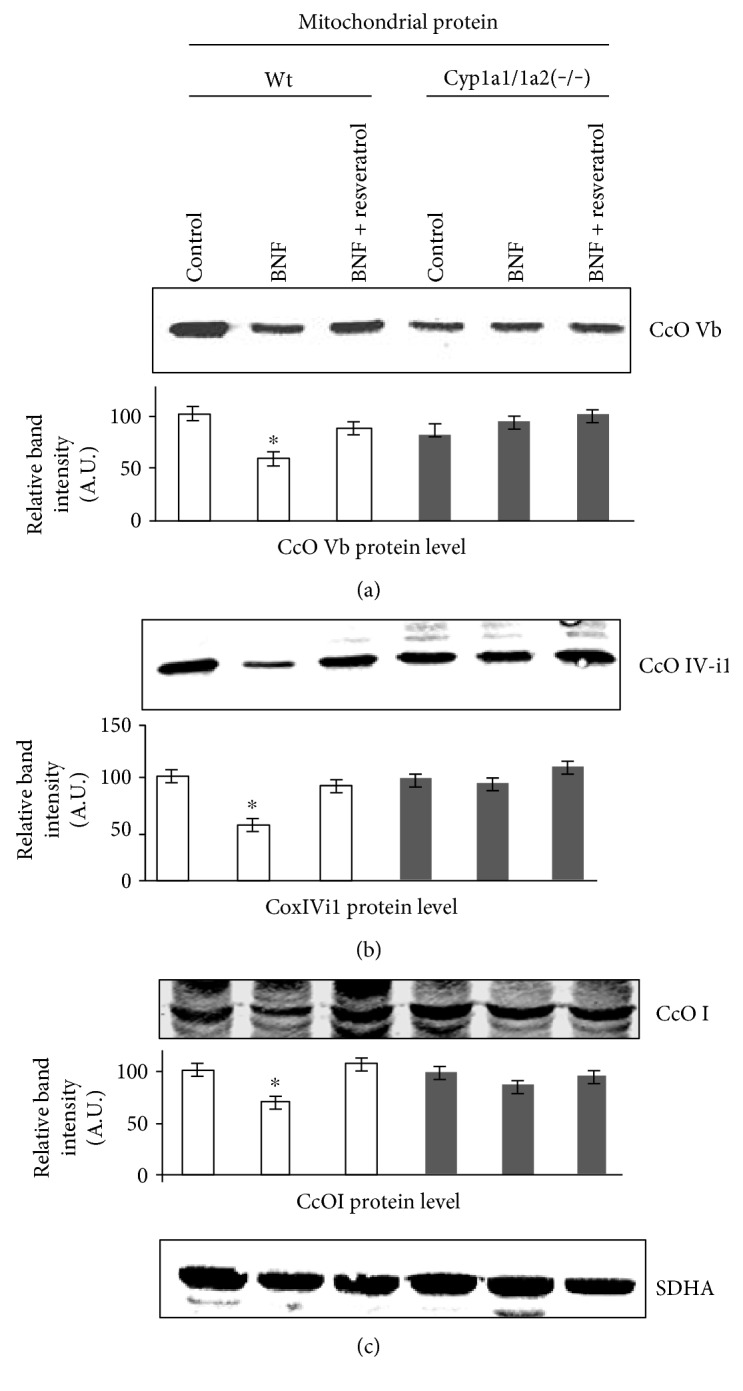
Effects of BNF and resveratrol on CcO subunit levels by immunoblot analysis. Immunoblot analysis of mitochondrial proteins from wild-type and double Cyp1a1/1a2(−/−) knockout, BNF-, and resveratrol-treated groups (*n* = 4 each). Mitochondrial proteins (50 *μ*g each) were resolved by SDS-PAGE on a 12% gel and the regions of blots corresponding to CcOVb, CcOIVi1, CcOI, and SDHA were excised based on apparent molecular masses and corresponding membrane strips were subjected to immunoblot analysis with anti-CcOVb (a), anti-CcO IVi1(b), and anti-CcOI (c) antibodies. Membrane strip corresponding to ~78 kDa was probed with antibody to the mitochondrion-specific marker succinate dehydrogenase (SDHA) as loading controls. The blots were imaged through a Li-Cor Odyssey Infrared Imaging System, and the band densities were quantified using the Volume analysis software. The values in the bar diagrams represent mean ± SEM of 4 independent experiment from different mice.

In previous studies, we found that CcO complex is an important biomarker for mitochondrial stress, since the levels of several subunits (I, IVi1, and Vb) were reduced under various stress conditions [34–36]. To assess the nature of BNF-induced changes in CcO complex, we assessed the levels of CcO subunits from control and *Cyp1a1/1a2(−/−)* mice. As shown in Figure 4, regions of the same blot corresponding to the indicated proteins were excised and probed with appropriate antibodies including CcO subunits Vb, IV-i1 and I, and also with antibody against SDHA. It is seen that subunits Vb (Figure 4(a)), IVi1 (Figure 4(b)), and CcOI (Figure 4(c)) levels were significantly reduced in Wt mice treated with BNF. Notably, resveratrol treatment in Wt mice restored the subunit level to near control level confirming the role of AHR-induced CYP1 genes in this loss. In contrast, BNF failed to affect the CcO subunit levels in *Cyp1a1/1a2(−/−)* mice and also resveratrol had no effect on these CcO subunits, confirming that BNF-induced CcO subunit loss requires the expression of CYP1A1 and 1A2 proteins. These results along with the results of Figures 3(a) and 3(b) suggest that the BNF toxicity targets complex I and IV of the mitochondrial electron transport chain. Results also show that resveratrol prevents the loss of enzyme activity as well as subunits of the CcO complex.

### 3.4. Effects of BNF and Resveratrol on *Cyp1a1/1a2* Gene and Protein Expression

**Figure 5:**
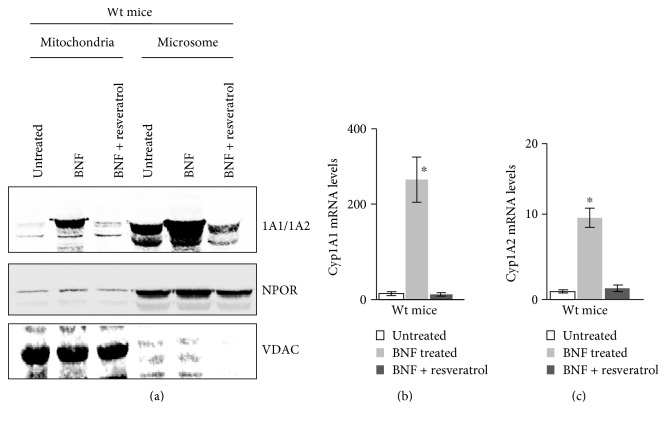
Effects of BNF and resveratrol treatment on CYP1A1/1A2 mRNA and protein expression levels. Immunoblot analysis of hepatic mitochondrial and microsome proteins from wild-type control, BNF-, and resveratrol-treated mice (*n* = 4each) following seven days of treatment. Proteins (50 *μ*g each) were resolved by SDS-PAGE on a 12% gel and subjected to immunoblot analysis with anti-CYP1A1/1A2 antibodies. Representative blot from one treated and untreated liver each has been presented. As described in Figure 4, all the three blocks of membranes were derived from the same blot. The blots were also probed with an antibody to the mitochondrion-specific marker voltage-dependent anion channel (VDAC) as loading controls (a). CYP1A1 (b) and CYP1A2 (c) mRNA levels were measured by real-time PCR in Wt control, BNF-, and resveratrol-treated mice as described in Materials and Methods. Results represent mean ± SEM from 4 independent experiments. ∗ denotes *p* < 0.05.

We tested the ability of resveratrol to inhibit BNF-mediated induction of CYP1A1 and 1A2 proteins in the liver mitochondria and microsomes by Western blot analysis. As seen from Figure 5(a), BNF induced high levels of hepatic mitochondrial CYP1A1/1A2 expression in Wt mice and more robust induction in the microsome. In both cases, resveratrol treatment attenuated BNF-mediated induction of CYP1A1/1A2. The level of NADPH-P450 oxidoreductase (NPOR) and mitochondria-specific marker voltage-dependent anion channel (VDAC) were used as markers for microsome and mitochondria, respectively. The levels of these markers show that the subcellular fractions used were devoid of significant cross-contamination. In agreement with results of the Western blot (Figure 5(a)), real-time PCR analysis of total liver RNA also showed that CYP1A1 mRNA was induced by >100 fold by BNF treatment, which was reduced to near control level in mice coadministered with resveratrol (Figure 5(b)). Similarly, CYP 1A2 mRNA was also induced by about 10-fold by BNF (Figure 5(c)). Coadministration with resveratrol effectively inhibited (<80%) BNF-mediated induction of both CYP1A1 and CYP1A2 mRNAs in mouse liver. As previously reported, resveratrol treatment on its own did not affect the expression of the CYP1A1 and CYP1A2 mRNAs [37].

### 3.5. Effect of BNF on ROS Production and Mitochondrial Respiratory Complexes in C6 Glioma Cells

**Figure 6:**
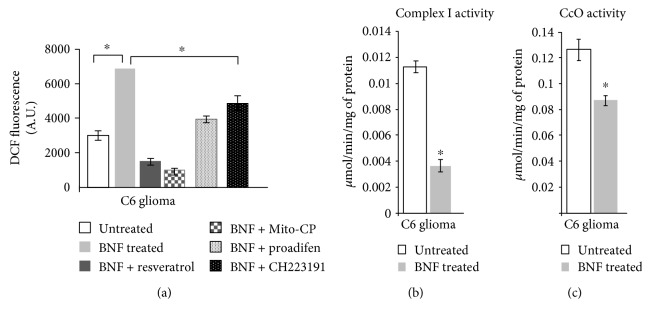
Effects of BNF on ROS production and mitochondrial respiratory complexes. ROS formation in C6 glioma cells treated with BNF, resveratrol, proadefin, or CG223191 was measured as described in Materials and Methods (a). Complex I assay was carried out using Cary 1E UV-visible spectrophotometer by incubating 10 *μ*g of freeze-thawed mitochondrial extract in 1 ml of assay medium. The assay medium consisted of 25 mM potassium phosphate, pH 7.4, 5 mM MgCl_2_, 2 mM NaCN, 2.5 mg/ml bovine serum albumin, 13 mM NADH, 65 *μ*M ubiquinone, and 2 *μ*g/ml antimycin A. The decrease in absorbance at 340 nm because of NADH oxidation was measured (b). CcO activity was measured by incubating 10 *μ*g of freeze-thawed mitochondrial extract from control and BNF-treated cells in 1 ml of assay medium (25 mM potassium phosphate, pH 7.4, 0.45 mM dodecylmaltoside, and 15 *μ*M reduced cytochrome c) (c). Results represent mean ± SEM of at least 4 independent experiments. ∗ denotes *p* < 0.05.

To further investigate the effects of BNF on ROS formation, we used C6 glioma cells expressing AHR-inducible CYP450s. As shown in Figures 6(a) and 6(b), ROS formation was significantly induced by BNF treatment, while resveratrol, and CH223191, both AHR antagonists CYP450 inhibitor proadifen, and Mito-CP treatment markedly inhibited BNF-induced ROS production, confirming the involvement of AHR-dependent CYP450 catalysis in ROS production. Mitochondrial respiratory enzyme activities were also measured in C6 glioma cells. Results show that the complex I enzyme activity was inhibited by about 60% (Figure 6(b)) while the complex IV (CcO) activity was inhibited by about 30% in BNF-treated cells (Figure 6(c)) when compared to untreated control.

**Figure 7:**
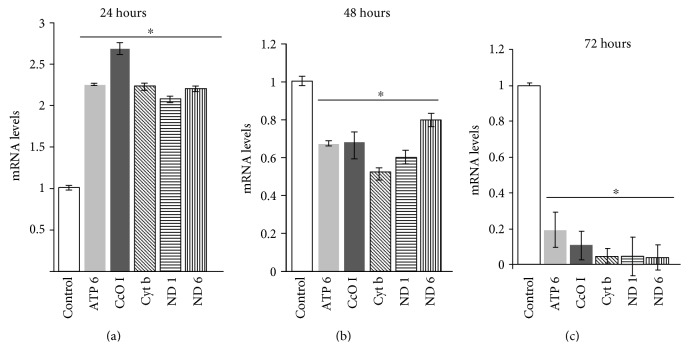
Effects of BNF on the expression of mitochondrial and nuclear genome-coded respiratory genes in C6 glioma cells. Mitochondrial genome-coded mRNA levels for ATP 6, CcO I, Cyt b, ND1, and ND6 from BNF-treated C6 glioma cells treated for 24 hours (a), 48 hours (b), and 72 hours (c) were quantified by real-time PCR with *β*-actin gene as internal control as described in Materials and Methods. Results represent mean ± SEM of 3 independent experiments. ∗ denotes *p* < 0.05.

To elucidate the possible mechanism by which BNF affects mitochondrial respiratory controls, we measured the level of mitochondrial gene expression at different times of BNF treatment from 24 to 72 h in C6 glioma cells. The relative mRNA levels for mitochondrial genome-coded ATP6, CcOI, cytochrome b, ND1, and ND6 were significantly increased (about 2-fold) by 24 h of treatment (Figure 7(a)), modestly reduced by 48 h of treatment (Figure 7(b)) and markedly reduced by 72 h of treatment (Figure 7(c)). The increase in transcription at early time period of 24 h probably represents a compensatory response to drug-induced mitochondrial toxicity representing extensive fission and fusion for repairing mitochondria. Continued exposure to the drug, however, induces time-dependent inhibition. These results show that BNF elicits marked inhibition of mitochondrial DNA transcription past the 48 h treatment time. Taken together, our results show that increased ROS production and mitochondrial dysfunction may be associated with induced expression of the AHR-regulated *CYP1A1* and *CYP1A2* genes.

### 3.6. Effects of BNF and CYP450 Inhibitor on ROS Production in Mitochondria from Treated Livers

**Figure 8:**
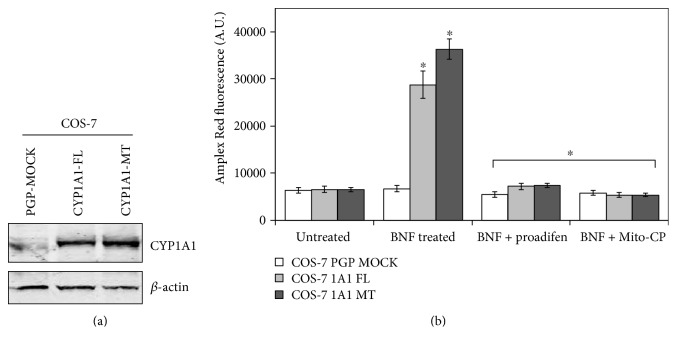
BNF-induced ROS production in COS-7 cells stably expressing CYP1A1 protein. COS-7 cells stably expressing full-length and N-terminal truncated (+33) CYP1A1 was generated as described in Materials and Methods. (a) Immunoblot shows the level of CYP1A1 expression in the two cell lines expressing full-length and +331A1 proteins. Total cell extracts (50 *μ*g protein each) were resolved on 12% polyacrylamide SDS gel, and the proteins were transferred to membranes as described in Materials and Methods. The blot was probed with antibody to rat CYP1A1 (1 : 1000 dilution) and *β*-actin. (b) ROS production as measured by Amplex Red method. Proadifen and Mito-CP were added as described in Figure 7. The immunoblot pattern in A is reproducible in multiple runs. The values in (Figure 8(b)) represent mean ± SEM of 3 independent experiments. ∗ denotes *p* < 0.05.

Since studies with hepatic tissue from BNF-treated mice and C6 glioma cell point to the catalytic activity of CYP1A1 and 1A2 as possible sources of ROS, we used COS cells stably expressing CYP1A1 to further ascertain this possibility. Immunoblot in Figure 8(a) shows the level of expression of full-length (microsomal) and N-terminal truncated (+33) (mitochondrial) CYP1A1 in COS-7 cells. Several previous studies ([1, 2]; Dasari et al. (2010); [38]) showed that N-terminal truncated CYP1A1 is more predominantly targeted to the mitochondria. The results of ROS measurements in Figure 8(b) show that BNF treatment induced ROS production in both Wt CYP1A1 expressing and +33-1A1 expressing cells. Proadifen, an inhibitor of CYP, and also Mito-CP markedly inhibited ROS production. These results confirm that the catalytic cycle of CYPs induce ROS production and much of the ROS produced is of mitochondrial origin.

## 4. Discussion

The toxic effects of TCDD and PAHs are thought to be mediated via AHR signaling [39]. Structural similarity of BNF, a flavonoid compound present in edible plants and fruits, with many PAHs and their respective amines make it a powerful activator of AHR, which in turn induces CYP1 genes along with an array of genes involved in inflammatory response ([14, 25, 39–41]). However, the mechanism involved in PAH toxicity is far from clear, and pharmacological tools have been routinely utilized to establish a cause-effect relationship between AHR activation and toxicities. Unlike the large number of PAHs from industrial sources whose metabolic products form adducts with DNA and induce mutations, BNF is not a mutagen because its metabolites are not highly reactive. Despite its suspected nonmutagenic and noncarcinogenic properties, reports suggest that BNF may affect some aspects of early development and cell differentiation [42]. In this study using *CYP1a1/1a2−/−* (double knockout) and *CYP1a1/1a2/1b1−/−* (triple knockout) mice as well as cell culture models, we show that BNF induces liver mitochondrial respiratory defect which is mediated through activated AHR and metabolic activities of AHR-induced CYP1A1 and 1A2 enzymes. Our results show that BNF treatment inhibits hepatic mitochondrial OCR by about 30–50% in wild-type mice. In contrast, in both double KO and triple KO mice, the BNF-induced inhibition of OCR was only marginal. Furthermore, the difference between the double KO and triple KO was not significant. This is consistent with several reports showing very low or no detectable CYP1B1 expression in rodent livers. Results also show that state 3 respiration, ATP-coupled respiration, as well as maximum uncoupled respiration was affected by BNF treatment. The reduced respiratory capacity is most likely associated with the inhibition of the activities of respiratory complexes, complex I and complex IV (CcO) (Figure 3). Similar results were also obtained when C6 glioma cells were tested under in vitro conditions. Results therefore suggest that inhibition of mitochondrial respiratory complexes by BNF may be mainly responsible for the observed mitochondrial dysfunction.

The induction of CYP1A1 mRNA and resulting enzyme activity has long been used as a sensitive indicator of AHR activation in numerous *in vitro* and *in vivo* models for screening a variety of compounds and environmental toxicants [43]. A strong correlation between AHR-binding affinity of the promoter sites, CYP1A1 induction, and dioxin-like toxicity of structurally related PAHs has been used as a biomarker for hazard identification and risk assessment of environmental pollutants, industrial chemicals, and therapeutic compounds [43, 44]. The present study also points to novel aspects of mitochondrial respiratory controls and electron transfer complexes as possible targets of AHR. In this study, we show that BNF treatment not only inhibits respiratory controls but also mitochondrial transcription in a time-dependent manner. The immediate effects of BNF at early time point (24 h) is increased transcription which is probably a compensatory effect of reduced electron transport chain activity. At 48 and 72 h of treatment, there is a time-dependent inhibition of mtDNA transcription. With a view to understand the molecular basis of this inhibition, we also measured ROS production and mitochondrial function in C6 glioma cell culture system which expresses CYP 1 genes. Our results show high level of ROS production accompanied by reduced mitochondrial complex activities in C6 glioma cells treated with BNF confirming the *in vivo* animal study on KO mice. Using Ahr and CYP specific inhibitors CH223191 and proadifen, respectively, we also demonstrated that ROS production was markedly inhibited suggesting the role of Ahr-dependent CYP activities in BNF-induced ROS production in the cell system. Some studies suggest that CH223191 is a more potent Ahr receptor antagonist against dioxin treatment in comparison to BNF [45]. In our cell systems, however, we found that resveratrol is equally or more potent antagonist of AHR based on CYP1A1 gene expression and ROS production.

We suggest that mitochondrial ROS production is most likely the cause of reduced respiratory enzyme activities and inhibition of mtDNA transcription. In this study, our results imply that CYP1A1 and 1A2 together induce mitochondrial dysfunction which might be critically important for reported toxic effects of BNF. A previous study from Nebert's group showed that microsomal CYP1A1 plays an important role in the elimination of BaP from the hepatic tissue for reducing xenotoxicity [21, 22, 38, 46]. Similarly, a previous study from our laboratory showed that TCDD induces mitochondrial dysfunction and retrograde signaling by directly acting on mitochondrial membrane complexes rather than through AHR activation [23]. In this respect, the two xenobiotics elicit mitochondrial toxicity through different mechanisms.

Resveratrol is a naturally occurring polyphenolic compound that occurs in grapes, peanuts, berries, and a number of plants used in traditional Asian medicine [47, 48]. This compound displays chemopreventive and several other properties useful for human health, including cardioprotective activity and inhibitory activity toward the ageing process. Several studies have reported that resveratrol inhibits the expression of a number of cytochrome P450 genes, including CYP1A1, CYP1B1, CYP1A2, CYP2E1, CYP2C8, CYP3A, and aromatase (CYP19) in cancer cell lines of different tissue origin in humans and other mammals, and also inhibits the catalytic activities of several of these CYPs, and it has been suggested that these inhibitions may underlie some of the cancer chemopreventive activity of this compound [49–53]. Several studies [27] suggest that low doses of resveratrol may help alleviate oxidative stress though its precise mechanism of action remains unclear. Using in vitro cell culture and in vivo mouse KO models, our results showed marked reversibility of mitochondrial toxicity by resveratrol. Our results with *CYP1a1/1a2−/−* double KO mice also show that the metabolic activities of mitochondrial CYP1A1/1A2 may be critical factors in inducing mitochondrial ROS production and associated mitochondrial dysfunction. Results with stable cells expressing Wt and N-terminal truncated CYP1A1 further support this possibility.

## Abbreviations

ADX: Adrenodoxin
ADR: Adrenodoxin reductase
AHR: Aryl hydrocarbon receptor
BNF: *β*-naphthoflavone
BaP: Benzo[*a*]pyrene
CcO: Cytochrome c oxidase
CYP: cytochrome P450
NPOR: NADPH-cytochrome P450 oxidoreductase
DCFDA: 2′,7′-dichlorofluorescin diacetate
mtDNA: Mitochondrial DNA
OCR: Oxygen consumption rate
ROS: Reactive oxygen species.
